# Is percutaneous Achilles tendon lengthening safe and effective for older children with idiopathic toe walking?

**DOI:** 10.1016/j.jposna.2024.100021

**Published:** 2024-02-28

**Authors:** Stephanie T. Kha, Halle D. Freiman, Danika Baskar, James G. Gamble, Steven L. Frick

**Affiliations:** Department of Orthopaedic Surgery, Stanford University School of Medicine, Division of Pediatric Orthopaedic Surgery, Stanford Children’s Health, Redwood City, CA, USA

**Keywords:** Idiopathic toe walking, Achilles tendon contracture, Percutaneous Achilles tendon lengthening

## Abstract

**Background:**

Treatment of older patients with idiopathic toe walking (ITW) can be challenging. Percutaneous Achilles tendon lengthening (pTAL) restores a normal gait pattern for younger children with ITW, but outcomes for older children are unclear. Concern exists that Achilles tendon lengthening in older children may cause residual calf weakness.

**Methods:**

We retrospectively evaluated 19 ITW patients older than 10 years of age who were treated with pTAL from 2001 to 2022, 17 of whom had documented ankle equinus with the knee in both extension and flexion in the clinic and in the operating room under anesthesia. Primary outcomes were absence of surgical complications, restoration of ankle motion and normal heel-toe gait pattern, and the ability to voluntarily toe walk and heel walk. Secondary outcomes were residual symptoms, limitations in mobility, use of regular shoewear, and self-reported parent satisfaction with the surgical procedure.

**Results:**

Mean age at time of surgery was 12.6 years (range 10–17 years), and average follow-up was 30.3 months (range 11.3–129 months). Seventeen patients (89%) had an Achilles tendon contracture pre-operatively, while two patients had passive ankle motion to neutral position with the knee in flexion. At final follow-up, fourteen patients reported no involuntary toe-walking; five patients reported occasional involuntary toe walking. All patients could voluntarily walk on toes and heels, had active ankle dorsiflexion at or above neutral, and had returned to full unrestricted activity. Most patients wore regular shoes. All parents reported satisfaction with the surgical procedure.

**Conclusions:**

The majority of older ITW patients have an Achilles tendon contracture and pTAL is a safe and effective treatment for these older patients. The procedure restores a normal heel-toe gait with no subjective residual ankle plantarflexion weakness.

**Key Concepts:**

(1) Achilles tendon contracture can develop in Idiopathic toe walker (ITW) patients as they age and walk on their toes.
(2) Percutaneous achilles tendon lengthening (pTAL) is safe and effective in older ITW patients with or without Achilles contracture.
(3) The pTAL procedure in older ITW patients can help restore a normal heel-toe gait with no residual ankle plantarflexion weakness.

**Level of Evidence:**

IV

## Introduction

Idiopathic toe-walking (ITW) is a diagnosis of exclusion that is given to children older than 3 years who consistently walk on their toes and have no identifiable neurological, muscular, or metabolic abnormality [1], [2], [3], [4], [5]. ITW is present in ∼5% of children, and natural history studies suggest that ∼80% of children will spontaneously improve or completely resolve by the age of 10 years [2], [4], [5]. For younger children who do not spontaneously resolve, percutaneous Achilles tendon lengthening (pTAL) has been shown to be an effective treatment option with few complications [6], [7], [8], [9].

ITW is uncommon in children who are older than 10 years of age. It is unclear whether these children continue to walk on their toes because they have developed an Achilles tendon contracture or if they continue due to a habitual pattern. Furthermore, the safety and effectiveness of pTAL in older patients is unclear because of a concern for residual muscular weakness [3], [4], [6], [7].

The purpose of this study was to evaluate our older ITW patients to determine the frequency of Achilles tendon contracture, to evaluate the safety and effectiveness of pTAL, and to determine if residual ankle plantarflexion weakness was a functional problem.

## Materials and methods

After obtaining institutional review board approval, we searched our electronic health records to identify patients who had undergone pTAL and were between 10 years and 18 years of age at the time of surgery. To isolate the cohort of idiopathic toe-walking patients, we excluded patients with neuromuscular and metabolic diseases, patients with autism spectrum disorder diagnosis, and patients who underwent pTAL as part of a larger foot reconstructive procedure during the time of surgery. To be included in the study group, patients must have had follow-up at least 9 months after their surgery. To determine the presence of an Achilles tendon contracture before surgery, the attending surgeon performed the Silfverskiöld test and visually estimated ankle range of motion under general anesthesia to eliminate any conscious impediment to dorsiflexion [10].

We identified 38 ITW patients that had been treated during the interval from 2001–2022. Nineteen patients (13 male, 6 female) met the final inclusion criteria. Reasons for further exclusion in the other patients were less than 9 months of follow-up after surgery for 9 patients, incomplete pre-operative documentation in the electronic medical records for 7 patients, concomitant osteochondromatosis in the affected limb in one patient, prior gastrocnemius recession procedure in one patient, and revision pTAL in one patient.

All 19 included patients from the practices of two attending surgeons had been treated with pTAL as described by Hoke and modified by Hatt [11], [12]. All patients were placed in a post-operative walking short leg cast for 4 weeks, and continued weight-bearing after the cast was removed. Post-operative bracing and physical therapy were not routinely utilized. We recorded patient demographic data, pre-operative and post-operative ankle motion, any complications such as bleeding, wound infection, inadvertent tendon transection, skin adhesions to the tendon, pain or discomfort, and numbness [13], [14], [15]. Final change in ankle range of motion was calculated as pre-operative maximum equinus subtracted from post-operative passive dorsiflexion with the knee extended. Gait outcomes were determined by visual assessment from the latest clinical evaluation. We recorded the presence of a stance phase heel strike, forefoot clearance in swing, and foot progression angle [16], as well as the ability of the patient to voluntarily walk on their toes (maintain equinus ankle position throughout the gait cycle) and to walk on their heels (ability to maintain active ankle dorsiflexion above neutral throughout gait cycle). Parents and patient were asked if they were satisfied with the results of the surgical procedure, and if the patient used special shoes.

### *Data analysis*

Descriptive statistics were performed using Microsoft Excel (Version 16.72, Microsoft Corp., Redmond, WA) to characterize the study population and describe outcomes in terms of proportion of the study cohort.

## Results

Table 1 shows the patient baseline demographics, pre-operative characteristics, and post-operative outcomes. The average age of all patients at the time of surgery was 12.6+/− 1.9 years (range 10–17 years), and average time to follow-up was 30.3+/− 27.6 months (range 11.3–129 months). The majority of our ITW patients, 17 of 19 (89%), had a pre-operative Achilles tendon contracture as demonstrated by ankle equinus with the knee in both extension and flexion in the operating room under general anesthesia. The other two patients could be brought only to the ankle neutral position with the knee in flexion. Estimated pre-operative passive ankle motion with the knee extended ranged from 0 to 45 degrees of equinus (average 18+/− 12 degrees equinus) and with the knee flexed ranged from 0 to 30 degrees of equinus (average 14+/− 9 degrees equinus). It is important to note that clinical estimations of ankle range of motion are subject to variability by the clinician evaluation [10].

**Table 1:** Patient baseline demographics, pre-operative characteristics, and post-operative outcomes after pTAL.

Pre-operative information				Post-operative outcomes					
Age at surgery (years)	Gender	Pre-op equinus contracture	Pre-op magnitude of equinus (degrees)	Follow-up time (years)	Equinus contracture at follow-up	Post-op involuntary toe-walking	Symptoms	Post-op dorsiflexion knee extended (degrees)	Change in ankle motion (degrees)
10	M	-	0	4.6	-	-		15	15
11	M	+	10	1.4	-	+		5	15
11	F	+	20	1.6	-	-		15	35
11	F	+	10	2.3	-	-		15	25
12	M	+	35	3.7	-	+	Lateral foot Numbness	15	50
12	F	+	10	3.2	-	-		10	20
12	M	+	10	0.9	-	-		5	15
12	F	+	45	1.0	-	+	Heel pain	0	45
12	F	+	20	1.6	-	-	Outward rotation of foot	10	30
12	F	+	20	2.7	-	-		10	30
12	M	+	20	1.0	-	-	Outward rotation of foot	5	25
12	M	+	10	1.1	-	+		5	15
12	M	+	15	10.6	-	-		10	25
13	M	+	35	2.5	-	-	Ankle pain	5	40
13	M	-	0	1.1	-	-	Heel pain	15	15
14	M	+	10	1.1	-	-		15	25
16	M	+	10	3.8	-	+		10	20
16	M	+	10	2.1	-	-		15	25
17	M	+	45	0.9	-	-	Outward rotation of foot	5	50

pTAL, percutaneous Achilles tendon lengthening.

(+) denotes the presence of the variable, (−) denotes absence of the variable.

At final follow-up, the estimated post-operative passive ankle dorsiflexion ranged from 0–15 degrees above neutral (average 10+/− 5 degrees). Final change in ankle range of motion averaged 28+/− 11.7 degrees. Seventeen patients (89%) had a normal heel-toe gait pattern and a normal foot progression angle at latest follow-up. The patients had no major post-operative complications, but there were minor complications including symptomatic numbness, pain, gait changes, and residual occasional toe-walking. Four patients had occasional episodes of toe-walking, estimated by parents to occur approximately once per week. Three patients had a post-operative external foot progression angle greater than 25 degrees and reported occasional ankle stiffness; one of these patients demonstrated resolution of her initial post-operative external rotation gait to a normal heel-toe gait by latest follow-up. Three patients had occasional lower extremity discomfort - two of which involved heel pain after a day of long walking and one involved ankle pain unrelated to activity. One patient also reported diffuse numbness on the lateral aspect of the foot that was improving. Eighteen of the 19 patients wore regular shoes; one patient preferred “high-top” shoes. Table 2 lists the post-operative outcomes and proportion of study cohort based on response.

**Table 2:** List of post-operative outcomes and proportion of study cohort based on response.

Post-operative outcomes	(n = 19)	
	Yes (%)	No (%)
Involuntary toe-walking post-operatively	26%	74%
Report any foot pain	21%	79%
Report pain when walking on only toes or heels	0%	100%
Report any limitations to daily life	6%	94%
Able to voluntarily walk on toes without difficulty	100%	0%
Able to walk on heels only	100%	0%
Walk with a Heel-toe gait pattern	89%	11%
Return to sport activities	100%	0%
Wear regular shoes	95%	5%
Dorsiflexion above neutral with knee extended	100%	0%
Parental satisfaction with outcome of pTAL for their child	100%	0%

pTAL, percutaneous Achilles tendon lengthening.

All patients had been released to unrestricted physical activities. All parents reported that they were satisfied with the outcome of the pTAL procedure for their child.

## Discussion

Most children with ITW resolve their toe walking spontaneously or undergo treatment before they are ten years old [4]. Treatment options for persistent ITW include observation, casting, physical therapy, orthosis, and surgery [17], [18], [19]. Percutaneous Achilles tendon lengthening has been reported to restore normal ankle motion and gait in young children with ITW [6], [7], [8], [9], [20], [21]. However questions remain concerning the treatment of older patients. For instance, do older patients develop an Achilles tendon contracture as demonstrated by ankle equinus with the knee in extension and flexion [3], [4], [20], [22]? Is pTAL safe and effective in older patients with ITW [4], [5]?

In our study of nineteen ITW patients who were 10 years of age or older and undergoing pTAL, 17 of the 19 patients (89%) had an Achilles tendon contracture when examined under general anesthesia. Two could be brought to the neutral position with the knee flexed. Percutaneous Achilles tendon lengthening restored ankle motion and a heel-toe gait pattern in all patients. The procedure did not result in excessive ankle plantarflexion or ankle weakness post-operatively and patients were able to return to an active lifestyle without needing special prescription footgear or orthoses. All parents were satisfied with the outcome of the surgery and all patients returned to unrestricted physical activity.

Other investigators have reported the results of surgical treatment in children with ITW. Kogan et al. reported all 10 patients in their case series who underwent pTAL had complete resolution of idiopathic toe walking at time of phone call follow-up, which ranged from 3 months to 6.5 years post-operatively. Although the authors do not report the average age of patients in this limited case series, they concluded that no patients exhibited any loss of strength at follow-up and all parents reported satisfaction with the procedure [7]. Similarly, McMulkin et al. demonstrated objective improvement in gait parameters in all 14 patients (mean age of 9.3 years at time of surgery) in their case series at 11 years follow-up, although only 6 out of 14 of their patients underwent percutaneous TAL. The remainder of the patients underwent other procedures including open TAL or gastrocnemius lengthening [8]. A study of ankle kinematics by Hemo et al. did find that 20% (3/15) of their patients occasionally walked on toes but had a normal gait most of the time at 2.9 years follow-up, while the remaining 12 out of 15 patients had complete resolution of idiopathic toe-walking and no patients had clinically relevant plantarflexion weakness [6]. Important to note is the mean age of their patient sample was 9 years and that open TAL was performed in the majority of the patients (12/15), while percutaneous TAL was performed in only 3 out of 15 patients [6].

To the best of our knowledge, our study is the first to report the results of pTAL in an older cohort of patients, with an average age of 12.6 years (range 10–17 years) at the time of surgery. Furthermore, while most of our patients had an equinus contracture at time of surgery, none of the patients had functional calf weakness assessed by clinical exam post-operatively, including the two patients without a pre-operative Achilles equinus contracture.

The percutaneous approach to Achilles tendon lengthening is effective in correcting ITW without the burden of a longer scar from an open or extensile approach, while still producing predictable outcomes and minimal risk of surgical complications such as infection, bleeding, or nerve damage. None of our patients experienced any surgical complications related to the pTAL procedure, and no patients required re-operation. While four patients observed occasional toe-walking after the procedure approximately once per week, all four of these patients could be reminded by their parents to walk with a heel strike gait successfully, and all were observed in clinic to have a heel-toe gait and had the ability to heel-walk when instructed.

Another benefit of the percutaneous approach is that patients are allowed to weight bear immediately on their casts and are encouraged to continue walking until casts are removed at 4 weeks post-operatively, thereby promoting mobility throughout the recovery course. Our study also demonstrated high parental satisfaction after the procedure, a key component in shared decision making for pediatric patients. As has been noted previously by Westberry et al. and McCulkin et al., [8], [21] Achilles tendon lengthening and correction of equinus (with associated inversion/internal rotation of foot) can uncover existing external tibial torsion, and we had three patients who walked with an outward foot rotation (observed as external foot progression angle) during walking after pTAL.

It is the authors’ experience that older patients may take longer to achieve a smooth heel-toe gait pattern than younger patients. They may benefit from a post-operative structured exercise program that focuses on gait training and tibialis anterior strengthening. Some patients may also experience foot discomfort or pain post-operatively. In our study, three patients reported occasional heel or ankle discomfort. The two patients with heel pain experienced discomfort after a day of long walking, and this pain improved after rest. The patient with ankle pain did not have activity-related pain and was referred for rheumatologic workup. While the cause of these symptoms is unclear, parents and patients should be informed that it is not uncommon for patients to have occasional heel pain that responds to a brief period of activity restriction.

This study has weaknesses including the retrospective nature of the study, lack of a comparison or control group, and relatively short follow-up. Clinical assessment of ankle motion and plantarflexion weakness was usually recorded in the charts by attendings based on visual inspection and estimation rather than with a goniometer for each measurement, and is thus subjective and variable. Telephone follow-up can also introduce recall bias for the reporter and the recorder. Also, we developed a questionnaire (Table 2) to assess outcomes felt to be important specific to the diagnosis and treatment of ITW, but did not use any validated PROMs.

## Conclusions

Achilles tendon contractures can develop in ITW patients, and pTAL is a safe and effective procedure for treating adolescent patients older than 10 years of age. Most patients had restoration of a normal heel-toe gait pattern, plantigrade feet, had returned to unrestricted activity, and did not require the use of orthoses or special shoes. All parents reported satisfaction with the outcome of pTAL.

## Availability of data and materials

The data that support the findings of this study are not publicly available due to containing information that could compromise the privacy of research participants.

## Additional links

• POSNAcademy: Achilles Tendon Lengthening by Sean Tabaie, MD
• POSNAcademy: 5-Minute Gait Analysis by Jon R. Davids, MD (2016 IPOS Preferred Techniques)
• POSNA Physician Education Study Guide: Gait

## Author contributions

**Kha Stephanie Tieu:** Data curation, Formal analysis, Writing – original draft, Writing – review & editing. **Freiman Halle D.:** Conceptualization, Data curation, Investigation, Methodology, Project administration. **Baskar Danika:** Conceptualization, Data curation, Investigation, Methodology, Project administration, Resources. **Gamble James G.:** Conceptualization, Data curation, Investigation, Resources, Supervision, Writing – review & editing. **Frick Steven L.:** Conceptualization, Formal analysis, Investigation, Methodology, Project administration, Resources, Software, Supervision, Validation, Visualization, Writing – review & editing.

## Ethics approval and consent

The authors obtained institutional review board approval for this study. The authors obtained informed consent from parents or legally authorized representative of the participants in this study.

## Declaration of competing interests

The authors have no disclosures and declare no financial interests related to this study.
